# The anatomical and imaging study of pes anserinus and its clinical application

**DOI:** 10.1097/MD.0000000000010352

**Published:** 2018-04-13

**Authors:** Sheng Zhong, Bo Wu, Miao Wang, Xiaohong Wang, Qi Yan, Xingyu Fan, Yanmei Hu, Yingying Han, Youqiong Li

**Affiliations:** a Department of Neurosurgery, the First Hospital of Jilin University; b Clinical College, Jilin University; c Department of Orthopedics, the First Hospital of Jilin University, Changchun; d Team of Student Brigade, Second Military Medical University, Shanghai; e Department of Respiratory Medicine, Jilin University First Hospital, Changchun; f Basic Medical College, Qiqihar Medical University, Qiqihar; g Department of Neurology, China-Japan Union Hospital of Jilin University; h Department of Human Anatomy, Norman Bethune Medical College, Jilin University, Changchun, China.

**Keywords:** anatomy, gracilis, pes anserinus, saphenous nerve, sartorius, semitendinosus, ultrasound

## Abstract

**Background:**

The pes anserinus was an important graft choice for anterior cruciate ligament (ACL) reconstruction. The infrapatellar branch of the saphenous nerve (IPBSN) might be damaged in this surgery. This study aimed to provide anatomic and ultrasonic measurement data of pes anserinus and superficial nerves.

**Methods:**

Eighty lower limb specimens of forty adult cadavers were dissected. The length, width, thickness, and the position of the tibial attachment of pes anserinus tendons were anthropometric measured, as well as the distance between the infrapatellar branch of the saphenous nerve and the pes anserinus. Sixty healthy adult participants were enrolled for ultrasonic research. The length, width, thickness of pes anserinus was also measured and the saphenous nerve was also assessed.

**Results:**

Anatomic results showed that there were 3 types of pes anserinus, the infrapatellar branch of the saphenous nerve (IPBSN) was almost paralleled to the upper edge of the pes anserinus tendon, and the average of distance between them was about 0.95 cm. The length of semitendinosus and gracilis tendons were 146.49 ± 12.83 mm and 124.62 ± 8.86 mm, the width of sartorius tendon was 25.58 ± 4.65 mm, wider than other tendons. The classification of pes anserinus tendons and the saphenous nerves could be identified in ultrasonic image. The length of semitendinosus and gracilis tendons were 151.35 ± 9.65 mm and 120.86 ± 8.99 mm, the width of sartorius tendon was 22.84 ± 3.83 mm. And there was no significance difference between anatomic and ultrasonic measurement (*P* > .05).

**Conclusion:**

The morphology of pes anserinus and its peripheral structures could be identified and measured precisely by ultrasound device, a presurgical ultrasonic examination was recommended. The arrangement of pes anserinus tendons was classified into 3 types according to our results. The incision should be performed medial to tibial eminence 1.5 cm and under the tibial tubercle level 2 to 3 cm, an oblique incision formed an angle of 50° with tibial transection was recommend, which was parallel to the direction of pes anserinus tendon.

## Abstract 2

Supplemental Digital Content is available in the text

## 1 Introduction

The anterior cruciate ligament (ACL) stabilizes the knee primarily by setting a limit to anterior tibial translation and rotational forces at the tibiofemoral joint. Anterior cruciate ligament rupture is one of the most common soft tissue injuries of the knee.^[1]^ Clinical manifestation of the patients with anterior cruciate ligament deficiency includes pain, increased instability, and altered function.^[2]^ In orthopedics, one of the current focuses is the surgery procedure in regard to the anterior cruciate ligament,^[3]^ the anterior cruciate ligament reconstruction is the major operation method to restore stability and function of the knee, as well as to lessen the likelihood of late degenerative disease.^[4]^ The reconstruction of anterior cruciate ligament has 2 essential processes as follows: first, an ideal implant should be comprehensively assessed, chosen, and obtained; then the fixture and fixed technology are performed to achieve solid therapeutic effects. Both 2 processes request surgeons a comprehensive and precise cognition about the anatomy of pes anserinus and popliteal fossa. With the rapid progress in medical technology, apparatus and instruments, the reconstruction surgery conducted with arthroscopy has improved a lot and now becomes a main-stream method to treat anterior cruciate ligament injuries.^[5]^

Both 2 types of transplants (including autografts and xenografts) have been used in anterior cruciate ligament reconstruction in previous researches, and the autografts are chosen preferentially since autografts are cheaper and more stable than xenografts.^[6,7]^ Generally, hamstring tendons are the preferred autografts for reconstruction of the anterior cruciate ligament, particularly gracilis and semitendinosus tendons which are collectively known as pes anserinus with sartorius tendon.^[8–10]^ The pes anserinus is an excellent graft choice and has become more popular for their robust strength, simple and convenient surgery procedure as well as a rather low morbidity of postoperative complications.^[11,12]^ Some studies evaluating treatment effects show that the quadruple-strand pes anserinus or patellar ligament grafts are even more potent to avoid failure with multiple fixation configurations when compared with native anterior cruciate ligaments biomechanically. It demonstrates a milestone triumph in grafts implanting surgery.^[9–12]^ In addition, achilles tendon, the latest implant for anterior cruciate ligament reconstruction, gets the increasing attention of current studies. However, there are still lots of limits when performed in the clinical practice, it has not been widely accepted by the most surgeons all over the world.^[13]^

Currently, there is few imaging research specifically focusing on the pes anserinus. Due to the lack of reports about the precise position of pes anserinus and its imaging characteristic, further improvement of the surgical quality is confined. Meanwhile there has not been an actually agreement that how operative incision performed will reduce complications when harvesting tendons for anterior cruciate ligament reconstruction. In addition, there are many complications after ACL construction, the infrapatellar branch of the saphenous nerve (IPBSN) or saphenous nerve may injury. With the increasing demand of less injury, an anatomic combined with imaging study illuminating pes anserinus, its peripheral structures and their relationships is necessary to be conducted. Ultrasound is widely used in anatomic study recently because of its real-time characteristic as well as its high-quality resolution regarding to soft tissue structures.^[14]^ Here, we conduct an anatomic combined with imaging study to elucidate and depict the morphology of the pes anserinus in both 2 measurements, as well as to provide applicable data for preoperative or intraoperative localization in regard of pes anserinus area.

## 2 Materials and methods

### 2.1 Materials

The anatomic dissections were performed at the Department of Human Anatomy, Jilin University. Eighty lower extremities specimens of 40 adult cadavers were dissected, 28 of which were male and 12 were female. All the specimens were xanthoderm, and their age ranged from 42 to 76 years, height ranged from 158 to 185 cm, and weight ranged from 44 to 85 kg. After checking body donation documents, we confirmed that no disease or past surgical incisions were noted in the areas dissected. All measurements were made by vernier caliper (minimum scale, 0.02 cm).

The ultrasonic protocol was performed at the Department of Ultrasound, First Hospital of Jilin University; it was approved by the ethics committee of First Hospital of Jilin University, and all participants were given written informed consent. We enrolled 60 healthy adult participants who were >18 years old and volunteered to participate in the study (38 female and 22 male; height range, 155–185 cm; weight range, 44–83 kg). All images were acquired by using an ultrasound device (GE LOGIQ E9 Ultrasound System [GE Healthcare, Fairfield, CT]) with a 10-MHz transducer for all the participants.

### 2.2 Procedure

**Figure 1:**
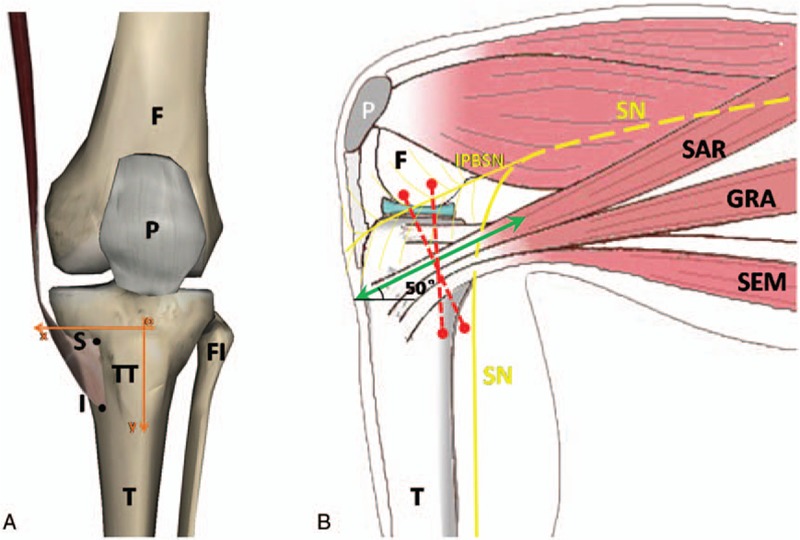
The diagram of pes anserinus, saphenous nerve distribution, and tibia. (A) F = femur, FI = fibula, I = inferior border of the tendon, P = patella, S = superior border of the tendon, T = tibial, TT = tibial tuberosity. *x*-Axis was the tibial crest and *y*-axis was the superior border of tibial tuberosity. The intersection of the *x*-axis and *y*-axis was designated as the origin point. Take point I (*x*_i_, *y*_i_) for example, *x*_i_, the horizontal distance between point I and tibial crest, *y*_i_, the vertical distance between point I and superior border of tibial tuberosity. (B) F = femur, IPBSN = infrapatellar branch of the saphenous nerve, P = patella, SN = saphenous nerve. Red dotted line noted the inappropriate or wrong incision which was not recommended (vertical incision or incision perpendicular to pes anserinus), green dotted line noted the appropriate incision direction which is formed an angle of 50° with tibial transection. GRA = gracilis tendon, SAR = sartorius tendon, SEM = semitendinosus tendon.

With the cadavers in the supine position, we made a longitudinal incision along the anterior mid-line of thigh and calf, and the overlying skin was removed to show the saphenous nerve and its branches. Then we observed and recorded the relationship of the saphenous nerve and the pes anserinus. After a careful deeper dissection of the pes anserinus, the tendons of semitendinosus, gracilis, sartorius were displayed from the tibial attachments at the tibial crest to the musculotendinous junction. We then observed the relationship of those tendons and measured the length, width, thickness and the tibial attachments of those tendons. The length of a tendon was defined as the distance from the tibial attachment to the musculotendinous junction, and the width of a tendon and the thickness of a tendon were measured at musculotendinous junction level. We also recorded the location of the tibial attachments by measuring the horizontal distance between the tibial attachments and the tibial crest, as well as the vertical distance between the tibial attachment and the superior levels of tibial tuberosity (as shown in Fig. 1A). The angle between pes anserinus and tibial horizontal transection was recorded by a protractor. The intersection between saphenous nerve and pes anserinus was also measured and located by the above methods. In addition, the infrapatellar branch of the saphenous nerve (IPBSN) traveled approximately parallel to pes anserinus, thus the distance between the infrapatellar branch of the saphenous nerve and the pes anserinus was also measured.

**Figure 2:**
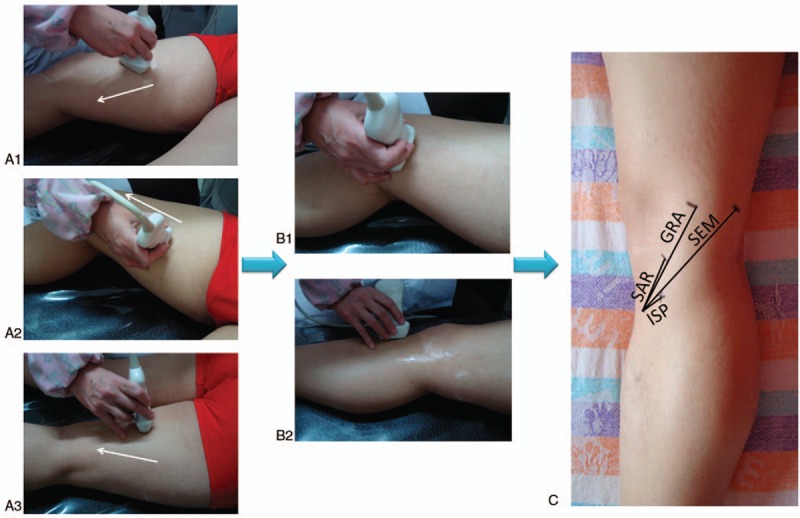
Procedures of ultrasonic examination. (A1–A3) Scanning the muscle of semitendinosus, gracilis, sartorius in the thigh, respectively. “White arrow” noted the direction of scanning. (B1) Scanning the pes anserinus tendons and saphenous nerve. (B2) Scanning the attachment point (tibial attachment) of pes anserinus on the tibia. (C) Measuring the length of pes anserinus tendons based on the signs marked on the skin. GRA = the length of gracilis tendon, ISP = the distance between the tibial attachment and the intersection formed by saphenous nerve and pes anserinus, SAR = the length of sartorius tendon, SEM = the length of semitendinosus tendon.

Ultrasonic measurements were taken while the participants were in a supine position. First, we scanned the sartorius, gracilis and semitendinosus muscles in the middle of the thigh respectively. Saphenous nerve was also easily identified by its typical characteristics: a low-echo screen mesh-like area at medial crus, branches and diverging sites were scanned carefully as well. Then we moved the probe along the muscle and tendon until the tibial attachments on the tibial bone. During this process, the tibial attachments, musculotendinous junction and the intersection between saphenous nerve and pes anserinus were all marked on the skin. Finally, the length, width and thickness of the semitendinosus, gracilis, sartorius tendons were measured, as well as the distance between the intersection and the tibial attachment of pes anserinus. We repeated examination procedures twice by the same technician to ensure consistency of the data^[15–19]^ (as shown in Fig. 2).

### 2.3 Statistical analysis

All the statistics were entered into SPSS 18.0 (SPSS, Inc., Chicago, IL) for analysis. The measurements were presented in the form of mean ± standard deviation. And we had normality texts for all the data. We performed an independent sample *t* text to measure the difference, with statistical significance set at *P* < .05.

## 3 Results

### 3.1 The morphology of the pes anserinus under anatomic observation

The pes anserinus consisted of semitendinosus, gracilis, and sartorius tendons at the anteromedial surface of the tibia. Among 80 lower extremities specimens of 40 adult cadavers, we found that the gracilis tendon inserted in the deep fascia which encompassed the sartorius, the tendons of gracilis and sartorius arranged compactly at the posteromedial corner of the knee joint, with the semitendinosus tendon accompanying at the posterior-lateral of the tendons of gracilis and sartorius. These tendons arranged in the order of the sartorius, gracilis, and semitendinosus tendons from anterior to posterior and from interior to inferior at the tibial attachment of pes anserinus. Furthermore, a few the sartorius tendons went over the tibial eminence and connected with contralateral periosteum in some specimens (4/80, existence ratio: 5%).

### 3.2 Classification of pes anserinus tendons arrangement

**Figure 3:**
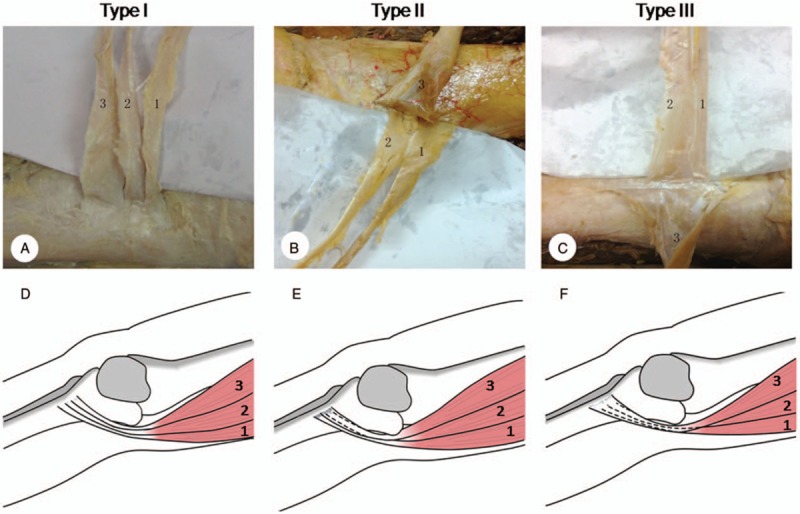
Anatomic image and diagram of pes anserinus classification. (A) and (D) Type I, (B) and (E) Type II, (C) and (F) Type III. 1 = semitendinosus, 2 = gracilis, 3 = sartorius.

We observed that the tendon of sartorius might cover gracilis and semitendinosus tendons at the tibial attachment of pes anserinus, and based on the coverage of the sartorius tendon we classified pes anserinus as follows: Type I (43.75%), the tendon of sartorius did not cover the tendon of gracilis, 3 tendons arranged abreast; Type II (15%), the tendon of sartorius covered gracilis tendon and incompletely covered semitendinosus tendon, it is the most rare type among the 3; Type III (41.25%), the tendon of sartorius covered gracilis and semitendinosus tendons completely (as shown in Fig. 3 and Table 1). Type I is the most common type among the 3 types.

**Table 1:**
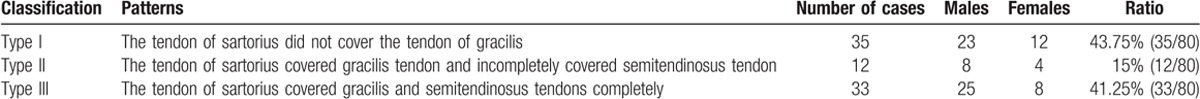
The classifications of pes anserinus tendons arrangement.

### 3.3 The anatomic measurement data of pes anserinus

Measurement results showed that the semitendinosus tendon was the longest tendon of pes anserinus with a length of 146.49 ± 12.83 mm, and the second longest one was the gracilis tendon, with a length of 124.62 ± 8.86 mm (shown in Table 2). These 2 tendons were considered as important grafts for anterior cruciate ligaments reconstruction. The length of sartorius tendon was 44.09 ± 4.29 mm, shorter than the 2 other tendons. The width of sartorius tendon was 25.58 ± 4.65 mm and it was wider than the width of gracilis or semitendinosus tendons (shown in Table 3). Hence, the sartorius tendon might cover the gracilis and semitendinosus tendons in some cases. The thickness of semitendinosus and gracilis tendons was 3.13 ± 0.41 mm and 3.07 ± 0.26 mm, respectively (shown in Table 4). The angle between pes anserinus and tibial tubercle transection was 50.46 ± 8.96° (ranged from 40.50° to 63.13°). In addition, we also measured and recorded the location of the tibial attachment of semitendinosus, gracilis, and sartorius tendons (shown in Table 5). The locations of the tibial attachments had great significance when determining the position, angle, and the length of incision in surgery.

**Table 2:**
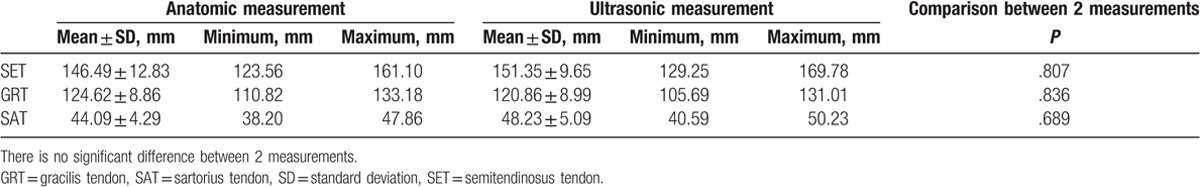
The anatomic and ultrasonic measurement data of the length of the hamstring tendons.

**Table 3:**
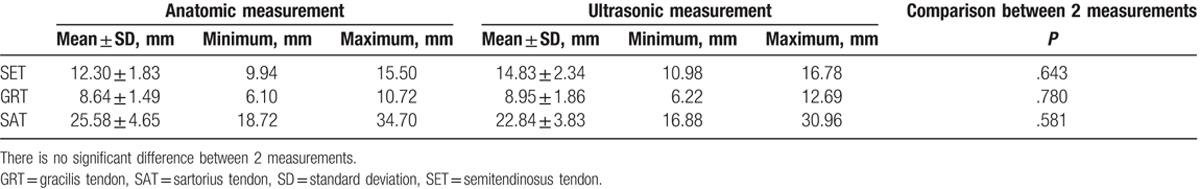
The anatomic and ultrasonic measurement data of the width of the hamstring tendons.

**Table 4:**
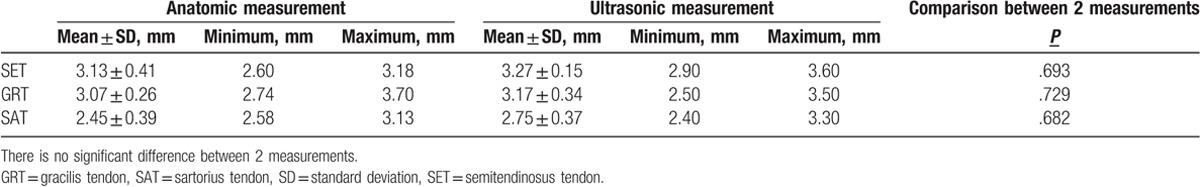
The anatomic and ultrasonic measurement data of the thickness of the hamstring tendons.

**Table 5:**
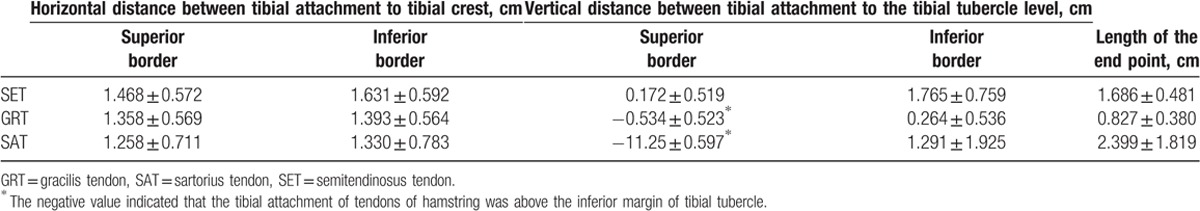
Anatomic measurement data of the tibial attachment of the hamstring tendons.

### 3.4 The ultrasonic measurement data of pes anserinus

**Figure 4:**
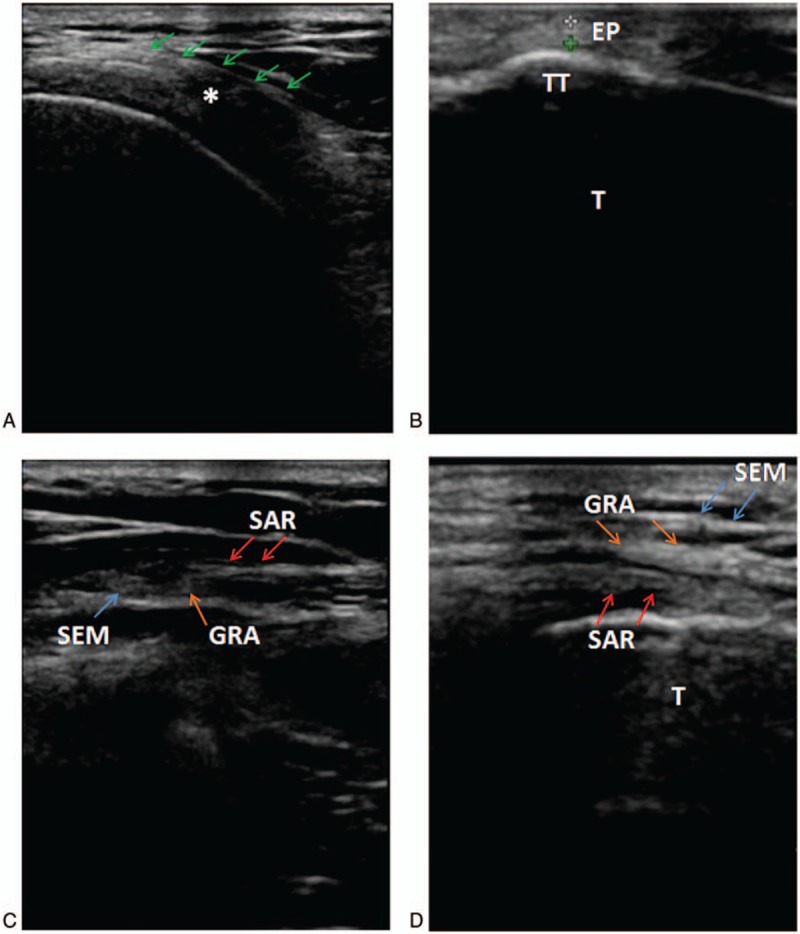
(A) ∗ = saphenous nerve, “green arrow” represented pes anserinus tendon; (B) EP = the tibial attachment of pes anserinus (among the white asterisk and green asterisk), T = tibia, TT = tibial tuberosity; (C) ultrasonic cross-section image. Type II arrangement of pes anserinus tendon: sartorius tendon covered gracilis tendon and incompletely covered semitendinosus tendon. GRA = gracilis tendon, SAR = sartorius tendon, SEM = semitendinosus tendon; (D) ultrasonic longitudinal section image. Type I arrangement of pes anserinus tendon: the tendon of sartorius did not cover the tendon of gracilis. (This image was scanned from the superior.) GRA = gracilis tendon, SAR = sartorius tendon, SEM = semitendinosus tendon, T = tibia.

The tendons of semitendinosus, gracilis, and sartorius were identified easily as a strong echo, elliptical area continuous with the muscles scanned by ultrasonic equipment. The peripheral structures and arrangement of these tendons could be detected, thus classification of pes anserinus tendon arrangement was determined by ultrasound, which was consistent with anatomic results. Tendons attachment point on the tibia was defined as the tibial attachment of pes anserinus, it is easy to identify (as shown in Fig. 4). Moreover, we could identify the saphenous nerve as a low-echo screen mesh-like area, which accompanied by the great saphenous vein at adductor canal. The result of ultrasonic measurement showed that the length of semitendinosus and gracilis tendons were 151.35 ± 9.65 mm and 120.86 ± 8.99 mm, respectively (as shown in Table 2). Sartorius tendon was the widest among the 3, the width of sartorius tendon was 22.84 ± 3.83 mm (as shown in Table 3). The thickness of semitendinosus and gracilis tendons was 3.27 ± 0.15 mm and 3.17 ± 0.34 mm, respectively (as shown in Table 4). And there was no significance difference in length, width, thickness of these tendons between anatomic and ultrasonic measurement (*P* > .05).

### 3.5 The distribution of the saphenous nerve

We found that the saphenous nerve at medial knee traveled between the gracilis and sartorius tendons and traveled through the deep fascia, the infrapatellar branch of the saphenous nerve (IPBSN) originated from the saphenous nerve and traveled approximately parallel to the upper edge of the pes anserinus. The saphenous nerve could be identified easily in ultrasonic image, while only a few IPBSN could be identified because of their minute size. The distance between infrapatellar branch of the saphenous nerve (IPBSN) and pes anserinus was 0.95 ± 1.26 mm in our anatomic study. And the anatomic results showed that the distance between the tibial attachment of pes anserinus and intersection formed by pes anserinus tendon and saphenous nerve were 7.38 ± 2.86 mm, and this distance was 6.99 ± 3.18 mm in ultrasonic measurement. There was no significance difference between them (*P* > .05) (as shown in Table 6).

**Table 6:**
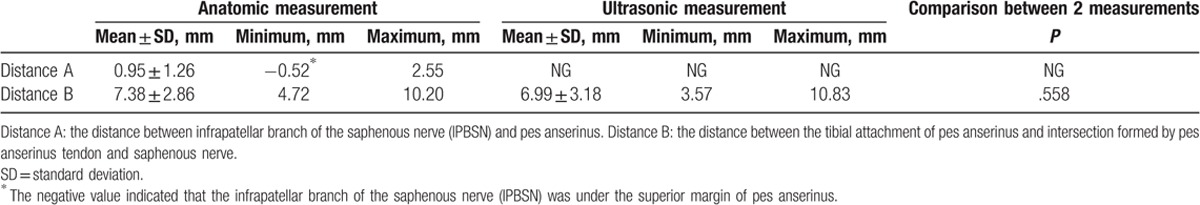
Anatomic and ultrasonic measurement data of saphenous nerve.

## 4 Discussion

The anterior cruciate ligament (ACL) lesion was a common soft tissue injury of the knee, with a high incidence and prevalence in sport activities. And there were many surgical techniques and graft choices had been provided for ACL reconstruction, such as patellar tendon, semitendinosus tendon, gracilis tendon, iliotibial band, quadriceps tendon, and various allogenic tendon grafts.^[20–23]^ In past decades, the central third of the patellar tendon and the pes anserinus had become the most common type of implants for anterior cruciate ligament reconstruction, because the operation for obtaining these tendons caused relatively lesser complications or demonstrable clinical functional impairment. Another reason to choose pes anserinus was that both semitendinosus and gracilis tendons could be harvested.^[24–26]^ Generally, sartorius tendon, with an average length of 4.4 m in our study, was too short to be chosen as implants. Recently, investigations had demonstrated that the muscle bulk might regenerate with time if sartorius tendon harmed after ACL reconstruction.^[27]^ On the other hand, the severe damage of sartorius tendon might incur certain dysfunction.

In addition, crural anteromedial skin sensation disorder might occur in some patients after anterior cruciate ligament reconstruction, because the dominating nerve of crural skin was saphenous nerve and its branches. The injury of the infrapatellar branch of the saphenous nerve was a common complication during harvesting semitendinosus and gracilis tendons process.^[28–30]^ The anatomic and ultrasonic location data of the pes anserinus and the saphenous nerve recorded in this study would give the surgeons a deepen comprehension about pes anserinus. Moreover, the ultrasonic locating procedure provided in this study also could be applied in clinical practice to avoid the injury of saphenous nerve.

The main conclusions of our study as follows: the morphology of pes anserinus and its peripheral structures could be identified and measured precisely by ultrasound device; the arrangement of pes anserinus tendons was classified into 3 types according to our results; the incision should be performed medial to tibial eminence 1.5 cm and under the tibial tubercle level 2 to 3 cm, an oblique incision formed an angle of 50° with tibial transection was recommend, which was parallel to the direction of pes anserinus tendon.

### 4.1 The clinical application of anatomic and ultrasonic measurement

The length, thickness, and width were measured and recorded in both anatomic and ultrasonic 2 methods. Among the 3 tendons, semitendinosus tendon was the longest and thickest tendon, it suggested that semitendinosus tendon was tough and stiff and might had a solid potential coping with great force. Gracilis tendon was medial long and thick, it could be graft as an ancillary role which assisted the semitendinosus tendon. Sartorius tendon was the shortest and the widest, such characteristic determined that it could hardly be a graft in ACL reconstruction. But it might be used in other plastic or reconstruction surgery. We also compared the results between anatomic and ultrasonic measurement, there was no significant difference between them. But the length and width in ultrasonic measurement was slight larger than that in anatomic. The reason might be that the cadavers were desiccated. The consistency between anatomic between ultrasonic measurement implied that ultrasound device had a potent ability to detect the morphology of pes anserinus and its peripheral structures. It suggested that ultrasound could also be used in diagnosing other pes anserinus relative disease, such as: anserine bursitis, pes anserinus cysts, or pes anserinus tendonitis.

We also found that the location of the tibial attachments of semitendinosus, gracilis, and sartorius tendons were relatively constant. The vertical distance from the inferior border of the tibial attachment of the semitendinosus and gracilis to the superior border of tibial tubercle was not exceed 4 cm, and the horizontal distance between the inferior border of the tibial attachment of these 2 tendons and the inferior border of tibial crest did not exceed 2 cm. The precise information about the tibial attachment of these tendons could be applied to locate the incision of harvesting tendons surgery. The incision should be performed medial to tibial crest 1.5 cm and under the tibial tubercle level 2 to 3 cm.

Furthermore, both the anatomic and ultrasonic measurements showed us that the length of semitendinosus and gracilis tendons were twice longer than the length of the anterior cruciate ligament (measured in previous studies), but the thickness of them were about half of the anterior cruciate ligament.^[16]^ Hence, it supported the argument that triple- or quadruple-stand pes anserinus grafts were an ideal choice for ACL reconstruction,^[17,31]^ for triple- or quadruple-stand pes anserinus grafts were almost the same size as anterior crucial ligament, it fit the place which were occupied by ACL previously.

### 4.2 The classification and the location of the tibial attachment of pes anserinus

In this study, the anatomic structure of pes anserinus and the arrangement relationship of the tendons of semitendinosus, gracilis, and sartorius were depicted in detail; the arrangement relationship of the pes anserinus tendons was classified into 3 types, which was firstly brought out by us. In previous researches, the structure of pes anserinus was roughly classified into 3 layers as follows: layer 1, the superficial layer surrounding the sartorius muscle; layer 2, the tibial collateral ligament; layer 3, the articular capsule of knee joint. But this kind of classification was vague and general, it did not depict the spatial location relationship of pes anserinus tendons and its clinical application was confined.

We suggested that surgeons should observe the arrangement relationship of the pes anserinus tendons carefully to avoid injuring the sartorius tendon when harvesting the tendons of semitendinosus and gracilis for anterior cruciate ligament reconstruction. And if the semitendinosus and gracilis tendons were partially covered (Type II) or completely covered (Type III) by sartorius tendon, surgeons should identify, separate, and free these tendons firstly, then retain the sartorius tendon as much as possible. In addition, tendon stripper should be used softer and carefully in case of postsurgical complications (functional disorder or sensational disorder). Nicholas reported that the semitendinosus might own 2 tendons or more,^[32]^ we also encountered such cases during our operation. The more tendons semitendinosus had, the thinner each tendon tended to be. In our opinions, all the tendons should be harvested to assure the toughness and stiffness of grafts.

### 4.3 The infrapatellar branch of saphenous nerve and direction of incision

The anatomic studies showed that the saphenous nerve at medial knee traveled between the tendons of gracilis and sartorius then through the deep fascia, and the infrapatellar branch of the saphenous nerve (IPBSN) originated from the saphenous nerve, which was consistent with previous studies.^[33]^ And we also found that the IPBSN was almost paralleled to the upper edge of the pes anserinus tendon, and the average of distance between them was about 0.95 cm (ranged from −0.52 to 2.55). It demonstrated a quite close distance from the IPBSN to the pes anserinus tendon, that is the reason why the IPBSN damage occasionally occurred during anterior cruciate ligament reconstruction. In addition, an inappropriate incision direction should also account for the IPBSN damage. Traditionally, the incisions of ACL reconstruction were relatively desultory, some surgeons preferred longitudinal incisions, while some surgeons preferred incision vertical to pes anserinus tendon, which was convenient to obtain tendons. However, based on our study, the 2 incision patterns mentioned above were all inappropriate, the most favorable incision should be an oblique incision which was parallel to the direction of pes anserinus tendon. Such incision would damage minimal nerve branches and avoid postsurgical complications as possible. Thus we suggested surgeons should perform an oblique incision, formed an angle of 50° with tibial tubercle transection, also parallel to the direction of pes anserinus tendon when harvesting graft to reduce the risk of the IPBSN damage (as shown in Fig. 1B). Regrettably, none of the IPBSN could be identified by ultrasonic device because of their minute size in our study, we suggest that the IPBSN may be identified and located to avoid being injured by other devices in the future.

Saphenous nerve emerged from the adductor canal and descended vertically along the medial side of the knee behind the sartorius. Along this process, the saphenous nerve encountered pes anserinus tendon, thus the intersection of these 2 structures was another key point. In our study, the intersection was shown clearly in ultrasonic image, saphenous nerve traveled beneath the pes anserinus or sartorius muscle, the distance between the tibial attachment of pes anserinus and intersection was 7.38 ± 2.86. In other words, surgeons should be careful to avoid damaging saphenous nerve when obtaining tendons or performing other surgeries in this area. In this study, the classification of pes anserinus tendon arrangement and distribution relationship of the sephenous nerves could be detected by ultrasonic equipment, which suggested that an presurgical ultrasonic examination could help surgeons to find out anatomic classification of the pes anserinus tendon and location of saphenous nerve, then corresponding signs should be marked on the skin. These examinations and procedures would reduce operation time, intraoperative infection rate, intraoperative blood loss and the risk of nerve damage or other complications. These results and conclusions are of great importance in clinical practice, it helps to consummate the current guidelines of ACL reconstruction.

## 5 Conclusion

The morphology (length, width, thickness, and tibial attachment) of pes anserinus and its peripheral structures can be measured precisely by ultrasound device. Ultrasound can be used in diagnosing pes anserinus relative disease. Among the 3 tendons, semitendinosus tendon is the longest and thickest tendon. Sartorius tendon is not suitable as grafts for ACL reconstruction. Our study supports the argument that triple-/quadruple-stand pes anserinus grafts are an ideal choice for ACL reconstruction. The arrangement relationship of pes anserinus tendons are firstly classified into 3 types. Type I (the tendon of sartorius does not cover the tendon of gracilis) is the most common type. Caution should be used to preserve the infrapatellar branch of the saphenous nerve (IPBSN) when harvesting tendons, the average of distance between pes anserinus and IPBSN is 0.95 cm. The arrangement relationship of the pes anserinus tendons and the intersection formed by pes anserinus tendons and saphenous nerve could be identified with ultrasonic device. The distance between the tibial attachment of pes anserinus and pes anserinus–saphenous nerve intersection was 7.38 cm. Surgeons should be careful to avoid damaging saphenous nerve when obtaining tendons or performing other surgeries in this area. A presurgical ultrasonic examination can clarify the pes anserinus peripheral structures and avoid damaging saphenous nerve. The surgery incision should be performed medial to tibial crest 1.5 cm and under the tibial tubercle superior border 2 to 3 cm, an oblique incision formed an angle of 50° with tibial horizontal transection is recommend, which is parallel to the direction of pes anserinus tendon to avoid the IPBSN damage.

## Author contributions

**Conceived the idea:** Y. Li, G.Z., and S. Zhong.

**Wrote the main manuscript text:** S. Zhong, B. Wu, and M. Wang.

**Checked and redressed the text:** Q. Yan and X. Fan.

**Analyzed the data and prepared the figures:** Y. Li.

**Reviewed the manuscript:** All authors.

## Supplementary Material

Supplemental Digital Content
