# The anatomical and imaging study of pes anserinus and its clinical application: Erratum

**DOI:** 10.1097/MD.0000000000010992

**Published:** 2018-06-01

In the article, “The anatomical and imaging study of pes anserinus and its clinical application”,^[1]^ which appeared in Volume 97, Issue 15 of *Medicine*, there are a few corrections that need to be made.

The corresponding author information should be “Yingying Han Department of Neurology, China-Japan Union Hospital of Jilin University, Street Xiantai 126, Changchun, China (E-mail: 17804312815@163.com).

The third to last sentence of section 4.2 should be “Noyes reported that the semitendinosus might own 2 tendons or more,^[32]^ we also encountered such cases during our operation.”

In the author contribution section, the authors who conceived the idea should be “Y. Li and S. Zhong.”

The following references should appear as:

[11] Atbasi Z, Ercin E, Erdem Y, et al. Correlation between body mass index and quadrupled hamstring tendon autograft size in ACL reconstruction. Joints 2016;4(12):2325967116674924.

[13] Noh JH, Kyiung HS, Yoon KH, et al. Supplementary tibial fixation in anterior cruciate ligament reconstruction—direct cortical fixation using spiked washer screw vs. post-tie using washer screw. Acta Orthop Belq 2016;82(2):358–364.

[15] Zhong S, Li G, Yang L, et al. Anatomical and ultrasonic study based on selective tibial neurotomy. World Neurosurg 2017;99:215–255.
