# Supplementary material for: The anatomical and imaging study of pes anserinus and its clinical application
Source: Medicine (Baltimore). 2018 Apr 13;97(15):e0352. doi: 10.1097/MD.0000000000010352 (PMC5908566; doi:10.1097/MD.0000000000010352)
Supplement: Supplemental Digital Content [file medi-97-e0352-s001.doc]

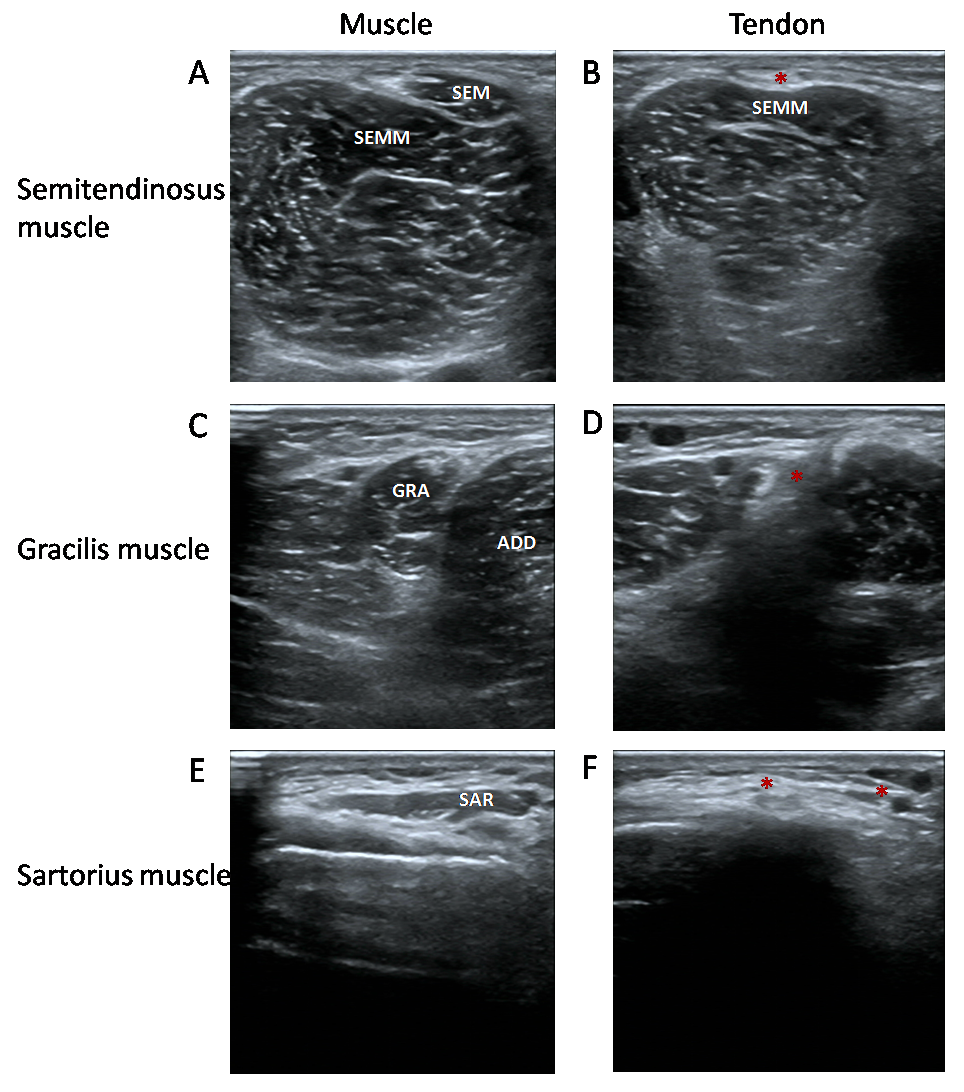


Appendix Figure 1. Ultrasonic image of muscles and tendons of pes anserinus. “SEM” represented semitendinosus; “GRA” represented gracilis; “SAR” represented sartorius; “SEMM” represented semimembranosus muscle; “ADD” represented adductor longus muscle. **(A)(C)(E)** displayed the muscles, while **(B)(D)(F)** displayed the tendons. **(A)(B)(C)(D)** were the ultrasonic transsection images, **(E)(F)** were the ultrasonic longitudinal section image. Red asterisk noted the tendons.
